# A Novel Approach for the Estimation of the Efficiency of Demulsification of Water-In-Crude Oil Emulsions

**DOI:** 10.3390/polym17212957

**Published:** 2025-11-06

**Authors:** Slavko Nešić, Olga Govedarica, Mirjana Jovičić, Julijana Žeravica, Sonja Stojanov, Cvijan Antić, Dragan Govedarica

**Affiliations:** Faculty of Technology Novi Sad, University of Novi Sad, 21000 Novi Sad, Serbia; nesic.22.21.d@uns.ac.rs (S.N.); jovicicmirjana@uns.ac.rs (M.J.); jzeravica@uns.ac.rs (J.Ž.); sonja.stojanov@uns.ac.rs (S.S.); antic.2.23.d@uns.ac.rs (C.A.); dragang@uns.ac.rs (D.G.)

**Keywords:** demulsifier, crude oil, W/O emulsion, separation efficiency, neural networks, response surface methodology

## Abstract

Undesirable water-in-crude oil emulsions in the oil and gas industry can lead to several issues, including equipment corrosion, high-pressure drops in pipelines, high pumping costs, and increased total production costs. These emulsions are commonly treated with surface-active chemicals called demulsifiers, which can break an oil–water interface and enhance phase separation. This study introduces a novel approach based on neural networks to estimate demulsification efficiency and to aid in the selection of demulsifiers under field conditions. The influence of various types of demulsifiers, demulsifier concentration, time required for demulsification, temperature and asphaltene content on the demulsification efficiency is analyzed. To improve model accuracy, a modified full-scale factorial design of experiments and the comparison of response surface method with multilayer perception neural networks were conducted. The results demonstrated the advantages of using neural networks over the response surface methodology such as a reduced settling time in separators, an improved crude oil dehydration and processing capacity, and a lower consumption of energy and utilities. The findings may enhance processing conditions and identify regions of higher demulsification efficiency. The neural network approach provided a more accurate prediction of maximum of demulsification efficiency compared to the response surface methodology. The automated multilayer perceptron neural network, with an architecture consisting of 3 input layers, 14 hidden layers, and 1 output layer, demonstrated the highest validation performance *R*^2^ of 0.991932 by utilizing a logistic output activation function and a hyperbolic tangent activation function for the hidden layers. The identification of shifted optimal values of time required from demulsification, demulsifier concentration, and asphaltene content along with sensitivity analysis confirmed advantages of automated neural networks over conventional methods.

## 1. Introduction

Chemical demulsification is the most commonly used method for separating water-in-oil (W/O) emulsions during crude oil dehydration. At the crude oil-gathering stations, this treatment is often performed at elevated temperatures [1,2,3,4,5,6,7,8,9]. The produced water has complex composition including high levels of salts, hydrocarbons, solids, and suspended particles. In the petroleum industry, multiple wells are connected to the gathering station and separation facilities, which can affect the variation in chemical composition and physical characteristics of dispersed water in the collected crude oil. In many investigations concerning chemical demulsification, a model emulsion was prepared using distilled water, sea water, tap water, or demi-water [10,11,12,13,14,15,16,17,18,19,20,21,22,23,24,25,26,27,28,29,30,31,32,33,34,35,36,37,38,39,40]. These model emulsions do not accurately represent field conditions because the produced water properties differ significantly from these fluids [10,11,12,13,14,15,16,17,18,19,20,21,22,23,24,25,26,27,28,29,30,31,32,33,34,35,36,37,38,39,40].

Statistical methods are valuable tools for addressing problems in the petroleum industry including the preparation of crude oil for transport, petroleum refining, or the separation of petroleum products. These methods have also been used in the demulsification of W/O emulsions [29,30,33,40,41,42,43,44,45]. In many studies, a central composite design (CCD) was employed to design experiments, and the analysis of variance (ANOVA) was used to predict the influence of variables on demulsification efficiency. Further, the response surface method (RSM) was used to predict demulsification efficiency and to determine optimal conditions [29,30,33,40,41,43,45]. These investigations revealed that an optimal demulsification efficiency exists as a dependent variable. The authors systematically analyzed demulsification based on various factors, including the nature of the demulsifier, demulsifier concentration, temperature, pH, oil/water ratio in the emulsion, settling time, etc. Despite many studies conducting the analytical analyses of demulsification efficiency, the influence of relevant variables and the sensitivity of statistical methods were often overlooked [40,41,42,43,44,45]. One major drawback is the necessity to conduct experiments with smaller sample sizes for such a sensitive process, increasing the likelihood of not capturing the optimal range for demulsification efficiency. For these reasons, a full factorial design paired with appropriate statistical methodology should be adopted to mitigate risk when working under high temperatures and concentrations, facilitating cost-effective chemical demulsification. Neural networks were investigated as promising tools in the petroleum industry for solving similar problems [46]. The investigation of neural networks as potential tools in the petroleum industry for solving similar problems has demonstrated their precision and reliability [1,47,48,49,50]. Additionally, they can detect complex nonlinear relationships between independent and dependent variables through various training algorithms.

Several interacting factors govern the stability of emulsions of water in crude oil, including droplet size distribution, salinity, pH, interfacial tension, total acid number, solids content, and shear history. However, practical monitoring at field gathering and dispatch terminals faces challenges due to operational constraints. Crude oil from multiple wells is collected through extensive, branched pipeline networks, making it difficult to obtain representative in-field droplet measurements. Additionally, reliable droplet sizing instruments, such as laser diffractors or advanced optical/acoustic analyzers, tend to be expensive, require frequent calibration and specialized maintenance, and are often unavailable at standard field terminals.

Furthermore, the processes of sample collection, transportation, and preparation can alter emulsion structure and skew laboratory measurements away from actual in situ conditions. For these reasons, this study emphasizes the use of routinely available operational predictors: temperature, demulsifier concentration, and settling time. It also incorporates a compositional proxy, asphaltene content, which was selected after SARA analysis, and sensitivity screening indicated that asphaltenes have the most significant impact on stability. Meanwhile, resins and waxes were found to have a minor influence. By limiting the set of predictors, we can also avoid overparameterization given the available sample size.

Despite the extensive use of response surface methodology (RSM) and ANOVA for screening demulsifiers, previous studies have primarily relied on simplified model emulsions, limited ranges of influencing factors, or small sets of experiments. This restricts their applicability to heterogeneous field crudes. Few investigations have utilized automated neural networks to predict the efficiency of demulsification for real produced-water emulsions, and none have systematically compared the RSM and multilayer perceptron (MLP) ensembles while quantifying prediction uncertainty and variable importance under full-factorial, field-relevant conditions. There is a clear need for predictive tools that can manage nonlinear interactions among demulsifier chemistry, concentration, temperature, time, and crude composition; provide robust sensitivity rankings to prioritize experimental efforts; and deliver actionable 3D response maps for operational decision making. This study addresses this gap by combining a modified full-factorial experimental design on field crude samples with automated MLP ensembles, global sensitivity analysis, and direct RSM comparison. The goal was to produce validated, uncertainty-aware models and provide practical guidance for demulsifier selection and process optimization.

The objective of this work was to introduce neural networks and the response surface methodology as statistical methods for estimating the demulsification efficiency of different commercial demulsifiers. The investigation encompassed the influences of a wide range of demulsifier concentrations, the time required for demulsification, and temperature on demulsification efficiency. The goal was to utilize neural network tools to reduce dimensionality and the number of variables, thereby improving the statistical evaluation of crude oil demulsification.

## 2. Materials and Methods

### 2.1. Materials

The crude oil emulsion was supplied from an oilfield in the northern region of Serbia. Crude oil properties were experimentally determined or estimated using standard methods. The density and API gravity of the crude oil were measured and calculated according to ASTM D4052 [51]. The pour point was determined following the ASTM D5853 [52] procedure. The SARA analysis was carried out based on the ASTM D6560 [53] and IP-368 [54] standards. The determined properties of crude oil are summarized in Table 1.

Crude oil is classified as heavy and medium paraffinic according to its density and paraffin content.

ISS Chemicals Company supplied five different demulsifiers, labeled as DEEM-1, DEEM-2, DEEM-3, DEEM-4, and DEEM-5. Table 2 gives the types of demulsifier.

**Figure 1:**
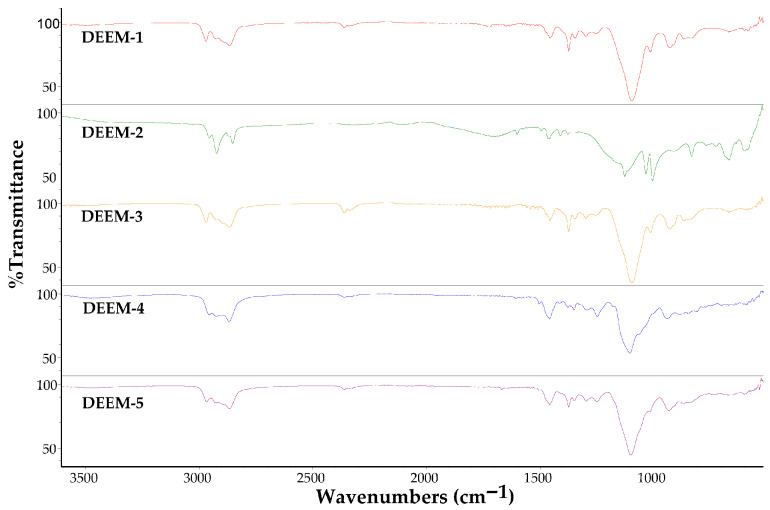
FT-IR spectra of investigated demulsifiers.

The infrared spectra of these demulsifiers were recorded using the Thermo Nicolet 5700 FT-IR spectrometer with attenuated total reflectance (ATR) FT-IR spectroscopy. The spectra were collected in the spectral range between 4000 cm^−1^ and 400 cm^−1^ with 1 cm^−1^ resolution (Figure 1).

### 2.2. Emulsion Preparation and Demulsification

All free water was removed from the crude oil sample. According to ASTM D4007 [55] test method, the oil emulsion contained 46 wt % of emulsified water. Hemos provided industrial-grade xylene for dilution, and all chemicals were used as-received without any further treatment.

The demulsification efficiency of each demulsifier was tested using a standard bottle test. All samples were placed in the laboratory water bath for one hour to obtain the designed separation temperature corresponding to oilfield processing conditions. After adding the demulsifiers, the bottles were shaken vigorously for 3 min before being returned to the laboratory water bath. Further, water separation was monitored for 12 h.

Standardized bottle tests were used with identically calibrated glass bottles. Samples were equilibrated to the target temperature before demulsifier addition, and bottles were shaken uniformly, then returned to the water bath. Separated water volume was read directly from bottle graduations at predefined time points and recorded. Replicate measurements were performed to assess reproducibility and compute measurement uncertainty. The oil–water interface was identified visually by contrast and by meniscus position in the calibrated bottle. In accordance with the petroleum industry standard bottle tests, the intermediate emulsion layer, a partially cleaned layer, is considered to be a part of the water phase. Anything entangled in the hazy, interfacial emulsion zone was considered as emulsified water and not free oil. In a bottle test, the main goal is to determine the amount of water that truly combines and separates into a completely distinct phase. By definition, everything that is still in the intermediate layer is still acting like an emulsion.

The volume of separated water was recorded as a function of time at specific time intervals. Demulsification efficiency (*DE*) was calculated by the following equation:

(1) $$DE=\frac{V}{V_{0}}\cdot 100(\%)$$

where *V* represents the volume of separated water, and *V*_0_ corresponds to the initial water cut.

### 2.3. Experimental Design

A modified full factorial design of experiments was employed to identify the optimal conditions for maximizing demulsification efficiency using the response surface methodology (RSM). The independent variables selected were temperature (*T*), demulsifier concentration (*C*), and time required for demulsification (*t*). The effect of these factors on the efficiency of demulsification (*DE*), which served as the dependent variable, was evaluated. The actual and coded values of the factors were determined, with the latter values corresponding to the low (−1), middle (0), and high (+1) levels.

Demulsification tests were conducted at five different demulsifier concentrations (20, 25, 30, 35, and 40 ppm) and four temperatures (36, 38, 40, and 42 °C) over a broad time range (0–12 h).

The experimental matrix of the modified factorial design included 240 experimental points for each demulsifier for the effect of process condition on demulsification efficiency and 60 experimental points for the effect of crude oil nature on demusification efficiency.

A modified full factorial design was selected instead of a central composite design (CCD) to ensure the comprehensive coverage of all factor combinations (temperature, concentration, time) and their higher-order interactions across the broad ranges encountered in field operations. This approach minimizes the potential extrapolation errors that can occur with a central composite design when investigating nonlinear regions of the demulsification process.

### 2.4. Statistical Analysis

The properties of samples collected at the oilfield gathering station were measured to develop an improved statistical tool for detecting the relationship between demulsification efficiency and relevant parameters. The investigations were carried out over a wide range of temperatures, concentrations, and times required for demulsification, corresponding to 1200 water-in-oil emulsion samples. The experimental data were analyzed using the response surface method (RSM) and neural networks. Statistical analysis was performed with the statistical package Statistica 14.0.0.15 (manufactured by TIBCO Software Inc., Palo Alto, CA, USA).

## 3. Results and Discussion

To select a demulsifier for treating water-in-crude oil emulsions and to design the corresponding equipment, it is essential to understand the physical and chemical properties of the crude oil and the available demulsifiers. In the petroleum industry, the rheology of a chemically treated emulsion is influenced by the location of the gathering station, which affects the handling of crude oil and water. The ratio of dispersed water in the emulsion may fluctuate and differ over time. Generally, the selected demulsifier should ensure reliable demulsification efficiency, optimal consumption, and a reduced time requirement for demulsification.

In this study, five different demulsifiers were investigated. The concentrations of the demulsifiers, their temperatures, and time required for demulsification were varied to determine the maximum demulsification efficiency. Furthermore, we propose a novel optimization approach based on neural networks. The results were analyzed using statistical methods, including 2D line plots and 3D surface plots, highlighting the advantages of the neural network approach over the traditionally used response surface methodology.

### 3.1. Response Surface Methodology

**Figure 2:**
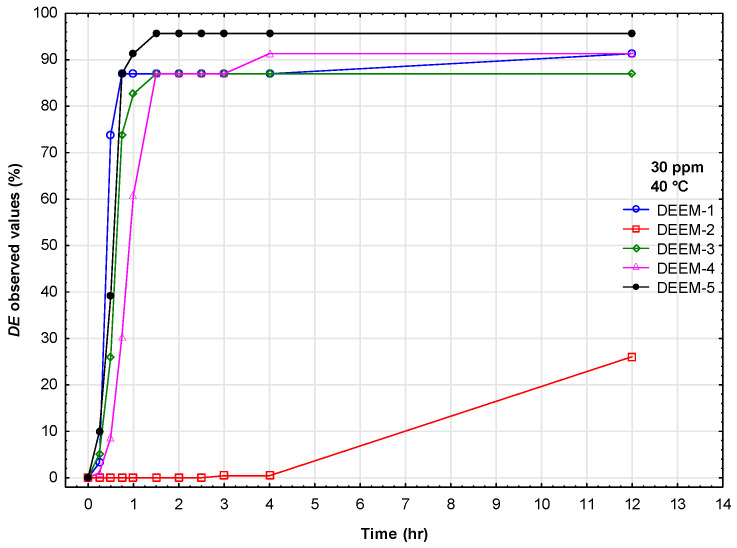
Dependence of demulsification efficiency on time required for demulsification for all demulsifiers (field conditions).

Each demulsifier was tested at five concentrations, four temperatures, and twelve time intervals. Since concentration and temperature may not remain stable over time, this investigation conducted preliminary demulsification tests using a bottle test in field conditions at 40 °C with a demulsifier concentration of 30 ppm. Figure 2 presents the relationship between demulsification efficiency on the time required for all demulsifiers under field conditions. DEEM-2 exhibited the lowest demulsification efficiency across all tested conditions. The benzenesulfonic acid headgroup in DEEM-2 imparts strong hydrophilicity, yielding a relatively high HLB value compared to the other alkoxylated demulsifiers (DEEM-1, DEEM-3–DEEM-5). In a water-in-oil (W/O) emulsion, effective demulsifiers must partition preferentially into the oil phase and accumulate at the oil–water interface. High polarity of DEEM-2 limits its oil-phase solubility and thus its interfacial adsorption, preventing it from effectively disrupting the rigid asphaltene/surfactant film that stabilizes water droplets. Furthermore, FT-IR analysis (Figure 1) highlights the strong S=O stretching band around 1100 cm^−1^ for DEEM-2, confirming extensive sulfonic functionality. While sulfonates can be excellent demulsifiers in oil-in-water systems, in W/O emulsions, they can often form reverse micelles in the aqueous phase rather than penetrate and fluidize the oil-side interfacial layer. Consequently, DEEM-2 fails to lower the interfacial tension sufficiently to promote the coalescence of water droplets. This contrasts with the more amphiphilic architectures of DEEM-1 and DEEM-5, which achieve balanced partitioning and efficient interface disruption.

The demulsifier DEEM-5 ensured the fastest water separation and the highest demulsification efficiency across the tested temperature and concentration ranges. DEEM-1 and DEEM-3 produced moderate performance with improved action at higher concentrations and longer settling times. DEEM-2 produced relatively rapid early separation but lower efficiency and greater sensitivity to temperature. DEEM-4 showed the lowest overall performance, especially at a low concentration and a low temperature. The demulsifier DEEM-5 combines an optimal hydrophile–lipophile balance and flexible polypropylene glycol chains that promote rapid adsorption at the oil–water interface and effective disruption of the asphaltene/resin stabilizing film. This interfacial activity accelerates droplet coalescence and yields higher demulsification efficiency under the tested field conditions. Figure 2 depicts the highest demulsification efficiency of the demulsifier DEEM-5 compared to other demulsifiers. Therefore, we conducted further investigations of this demulsifier using response surface regression and neural networks.

In this paper, a modified full factorial design was employed, allowing for a more reliable analysis with a larger number of experiments, which improved the optimization precision. The experiments utilized a statistical approach based on the response surface methodology, resulting in a polynomial second-order model. By applying the response surface methodology to the data collected on demulsification efficiency test results, the following equation was developed for DEEM-5:

*DE* = 472.1300 − 31.7754∙*T* + 24.5461∙*t* + 8.9790∙*C* + 0.4260∙*T*^2^ − 1.5904∙*t*^2^ − 0.1356∙*C*^2^ (2)

The test results for sum of squares (SS) comparing the entire model to the residual values are displayed in Table 3.

**Figure 3:**
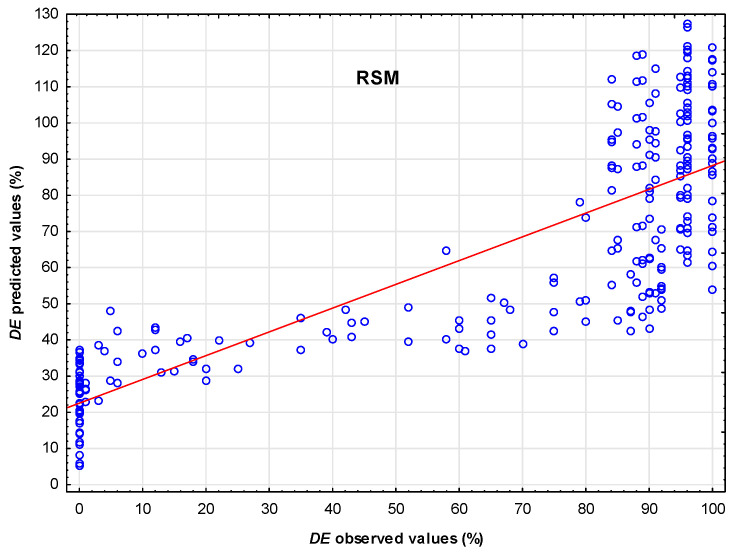
Observed versus predicted values for demulsification efficiency using RSM.

The calculated multiple *R* was 81.0701%, the multiple *R*-squared was 65.7236%, and the adjusted *R*-squared was 64.8410%. This test indicates a very strong correlation between the variables. Figure 3 demonstrates the deviations between the observed and predicted values of demulsification efficiency.

Figure 3 shows that the dependence of observed values on predicted values of demulsification efficiency, revealing that this relationship is not a linear correlation. The strong curvature and clusters can indicate issues with this regression model, which is commonly used to investigate demulsification efficiency in previous investigations.

**Figure 4:**
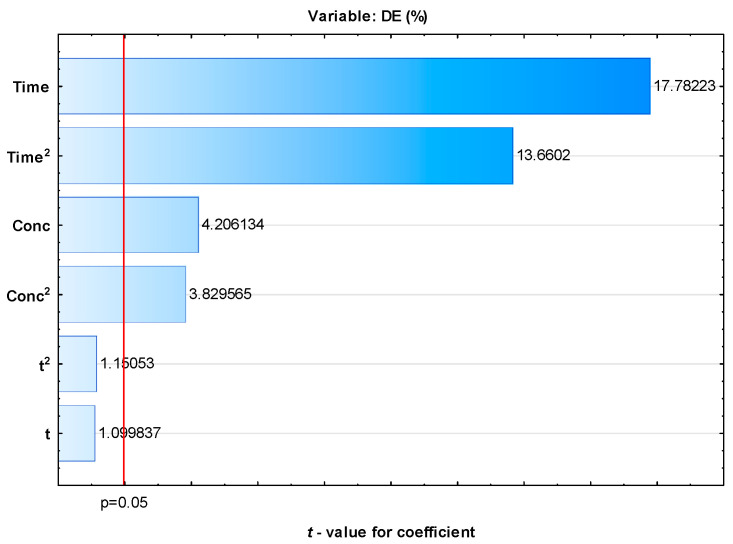
Pareto chart for standardized effects for *DE* calculated by RSM.

A Pareto chart of *t*-values for coefficients is shown in Figure 4.

**Figure 5:**
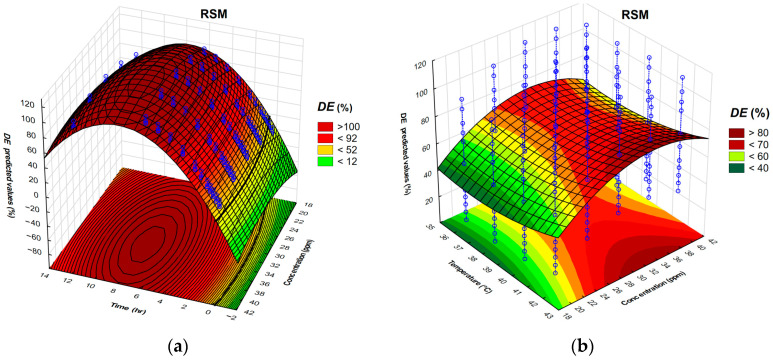
Three-dimensional plots representing the interdependence of demulsification efficiency on (**a**) time and concentration and (**b**) temperature and concentration.

Pareto chart for standardized effects for *DE* calculated by the RSM shows that the time required for demulsification and the concentration of the demulsifier are the most significant parameters affecting demulsification efficiency. Considering the accepted *p*-value limit of 0.05 or lower, the linear and quadratic terms of both time and demulsifier concentration had the greatest impact on demulsification efficiency. The demulsification analysis can be further enhanced by using 3D surface plots of the dependent variable (*DE*). Figure 5a illustrates the interactions among time, temperature, and the concentration of demulsifier DEEM-5 on demulsification efficiency.

Based on 3D surface plots, it can be concluded that there is an optimal region of time and concentration for efficient demulsification. This conclusion aligns with previous investigations [29,30,33,40,41,42,43,44,45]. The time required for demulsification, temperature, and concentration were varied in order to identify the maximum demulsification efficiency. The highest *DE* was achieved with a demulsification time of 7.5 h and a demulsifier concentration of 35 ppm (Figure 5a). The maximum of demulsification efficiency corresponds to the time required for demulsification of 7.5 h and the demulsifier concentration of 35 ppm (Figure 5a). The results from various temperatures and demulsifier concentrations reveal a narrow region of high *DE* (Figure 5b). One notable region is characterized by a demulsifier concentration of 32 ppm and a temperature of 35 °C, which yields nearly 75% of DE. Another region corresponds to the demulsifier concentration of 32 ppm and the temperature of 42 °C, obtaining 77% of *DE*. It should be noted that *DE* values estimated by response surface differ from *DE* values obtained on the oilfield, and these values can be significantly underestimated. As previously noted, real oil production often requires managing simultaneous changes in concentration and temperature. This scenario is particularly relevant in oilfield gathering stations during the winter, but similar investigations have not addressed this issue.

However, a great majority of investigations have been confined to a relatively narrow range of investigated variables; therefore, the findings were contradictory [29,30,33,40,41,42,43,44,45]. Generally, these approaches demonstrate the weakness of inappropriate experimental design. With a few experiments, the reliability of response surface regression can be greatly diminished. While the coefficient of determination for these models may be high, it is not the sole criterion for selection of demulsifier. Figure 5b demonstrates that the RSM surface does not include all the data points in a common field situation, including simultaneous temperature and concentration variation. The developed RSM is represented by a saddle-shaped surface, which fails to include all relevant data points. The deviation between the model DE and the saddle point DE indicates the non-reliability of the observed approach.

### 3.2. Automated Neural Network Model

To improve model accuracy, automated neural networks were used. Ten thousand networks were selected and trained using the multilayer perceptron neural networks (MLP) architecture.

Each neuron generates its output by performing a weighted sum of its inputs and then passing it via a transfer function *f*. In an MLP trained network, there is additionally a bias term for every neural layer. A bias is a neuron that has its activation function set to 1 constantly. Similar to other neurons, a bias uses a weight, also referred to as a threshold, to link to the neurons in the layer above. A layered feedforward topology is used to assemble the neurons and biases. Thus, the weights and thresholds are the free (adjustable) parameters of the model, and the automated neural network may be simply interpreted as an input–output model. The number of layers and units in each layer determines the function complexity, allowing such networks to simulate functions of nearly arbitrary complexity.

Random sampling was used as the sampling method, with 70% of data for training, 15% for testing, and 15% of data for validation. Furthermore, 1000 seeds were used for sampling. The number of hidden layers was from 3 to 15. From 10,000 trained *MLP*s, the top five were selected by ranking their validation *R*^2^ and validation *RMSE* on the hold-out set. Overfitting was mitigated by early stopping, terminating training when validation error ceased to improve, and by using three data splits (70% training, 15% testing, 15% validation). Ten thousand networks were trained and evaluated based on sum of squares as the error function. A total of five neural networks with the lowest error function (sum of squares) were retained.

Broyden–Fletcher–Goldfarb–Shanno (*BFGS*) or Quasi-Newton second-order training algorithm was used for training networks with very fast convergence but high memory requirements due to the storing of Hessian matrix.

The set of neuron activation functions for the hidden and output layer of neurons is given in Table 4.

The relative significance of the variables utilized in a neural network is investigated by global sensitivity studies. In sensitivity analysis, automated neural networks are examined as changing each of its input variables affects the neural network’s responses (predictions) and, consequently, the error rates. This approach enables the efficient quantification of input variable contributions to a model’s output across the entire input space. A global sensitivity analysis involves repeatedly submitting the dataset to the network, replacing each variable with its mean value derived from the training sample, and then recording the resulting network error. This error rises significantly if a significant variable is altered in this way; it does not rise much if a minor variable is eliminated. Interaction effects are captured implicitly by the MLP through its nonlinear mapping and network weights. The sensitivity indices quantify each input’s aggregate influence on output but do not separately decompose pairwise or higher-order interaction terms in an explicit algebraic form as the RSM does.

### 3.3. Effect of Demulsifier Nature on Demulsification Efficiency by Automated Neural Networks

**Figure 6:**
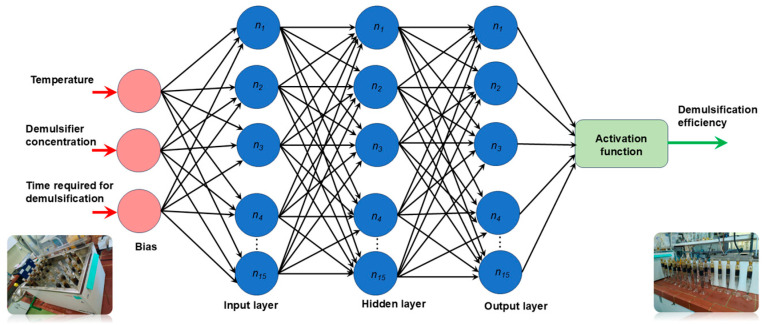
The architecture of automated neural network used for investigation of the effect of temperature, demulsifier concentration, and time required for demulsification on the efficiency of demulsification.

Figure 6 shows the architecture of automated neural network used for investigation of the effect of temperature, demulsifier concentration, and time required for demulsification on the efficiency of demulsification. This approach was conducted to study and predict the demulsification efficiency of the demulsifier DEEM-5 on one type of crude oil.

A summary of selected active networks is shown in Table 5.

A summary of active networks’ training algorithm and hidden and output activation is shown in Table 6.

A neural network MLP 3-14-1 was chosen due to its highest training, test, and validation performance. The neural network MLP 3-14-1 corresponds to the architecture of 3 input layers, 14 hidden layers, and 1 output layer.

Model selection was guided by the highest validation performance and lowest split-to-split variance. Although MLP 3-9-1 exhibited marginally higher test accuracy, MLP 3-14-1 delivered more consistent results across training, validation, and testing; thus, it was selected over an ensemble for practical deployment.

Quantitatively, the final *RSM* model achieved *R*^2^ = 0.657 and *RMSE* = 7.8% *DE*, whereas the *MLP* 3-14-1 achieved *R*^2^ = 0.991932 and *RMSE* = 1.4% *DE*. On a standard eight-core workstation, fitting the *RSM* polynomial required 3 s, while the full MLP search and training pipeline (10,000 networks) was completed in 8 min. Given the 80% reduction in prediction error, this computational method is justified for field screening.

**Figure 7:**
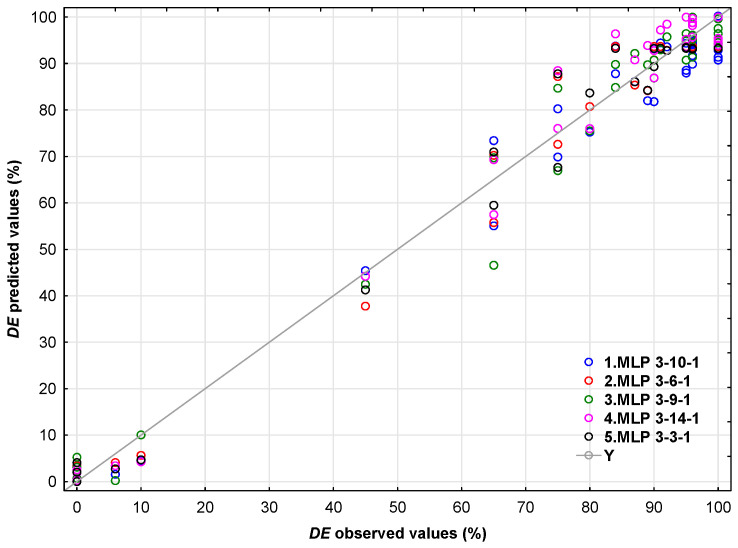
Relationship between the observed and predicted values of the *DE*.

The highest validation performance is observed for the logistic output activation function and hyperbolic tangent hidden activation. Furthermore, this neural network exhibited the lowest validation error. Figure 7 shows the deviations between the experimentally determined DE values (observed) and those predicted using the selected MLP 3-14-1 and other neural networks.

**Figure 8:**
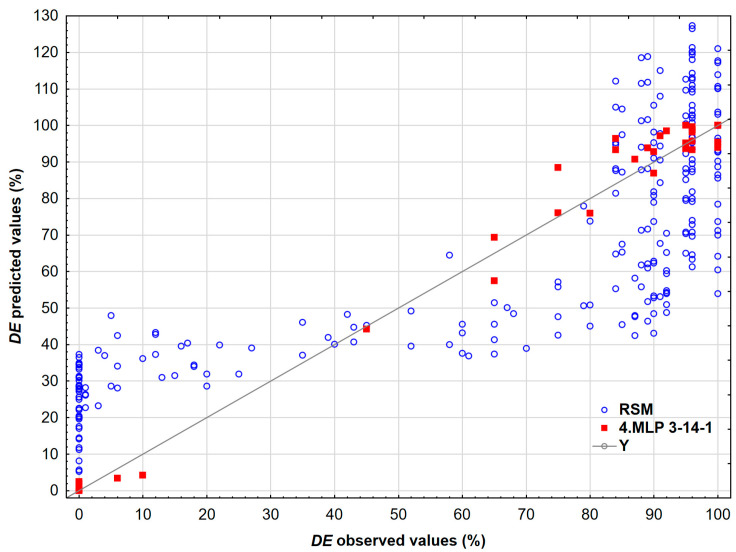
The comparison between predicted and observed *DE* values for the model developed by the neural network MLP 3-14-1 and RSM.

The scatter plot (Figure 8) compares the predicted and observed demulsification efficiency for the MLP 3-14-1 neural network and the RSM model. The results show that the predictions from the MLP 3-14-1 model cluster closely around the line *Y* = *X*, indicating a high level of accuracy. In contrast, the predictions from the RSM model demonstrate greater scatter and systematic deviations, which suggests that the neural network model has superior accuracy and lower bias.

All networks produced consistent large-scale surface topologies and identified the same high-efficiency regions, with only slight differences in peak locations. The quantitative differences were minimal: the Root Mean Square Error (*RMSE*) and *R*^2^ values across the retained models varied only slightly, and the ensemble mean predictions closely matched the experimental values. Global sensitivity rankings remained stable across models, with the order being time > concentration > temperature in all cases, and relative importance values differed by no more than 15%. Minor local variations in gradient steepness and the sharpness of optimal points reflect alternative internal parameterizations rather than contradictory physics. These results indicate that multiple networks can achieve comparable predictive performance while utilizing different internal logics, yet they converge on the same practical recommendations.

**Figure 9:**
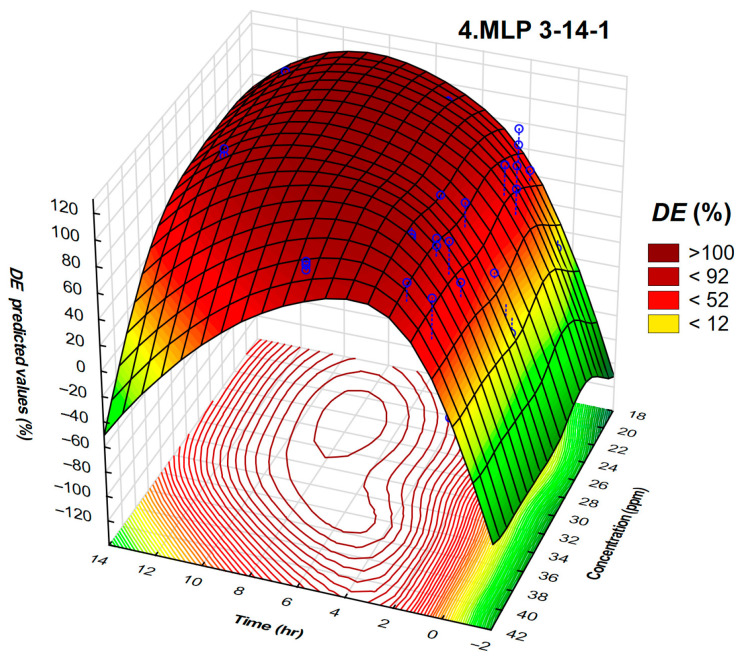
A three-dimensional plot representing the interdependence of the demulsification efficiency, time, and concentration of the demulsifier DEEM-5 for neural network 4.MLP 3-14-1 validation.

Each dot corresponds to one bottle test result. This approach can be used to predict the *DE* for a wide range of temperatures, concentrations, and time. A visual inspection of the presented approach reveals that the data are randomly spread around the regression line, implying no systematic lack of fit. It is clear that neural networks adequately fit measured *DE* values. Interactions between the time and concentration of the demulsifier DEEM-5 on the demulsification efficiency using neural networks are shown in Figure 9.

The region of the highest *DE* is shifted to a section of lower time required for demulsification. This was not possible to detect using the response surface methodology. This 3D plot shows, for the demulsifier DEEM-5, the relationship between the effect of time and concentration on demulsification efficiency. Demulsification efficiency at shorter time intervals is low. The region of the high *DE* on the plot corresponds to the time required for demulsification of 4.5 h and a concentration of 36 ppm. Neural networks improved the model’s accuracy, clearly showing the optimal values zone on the 3D surface. Lower values of time required for demulsification can contribute to increased crude oil processing rate and lower processing costs. Furthermore, neural networks revealed the region where DE is practically independent of the demulsifier concentration. This approach could increase the energy efficiency of crude oil processing, including reducing the settling time in separators, improving crude oil dehydration, increasing processing capacity, and lowering energy and utilities consumption (steam, heat, chemicals, etc.). In some cases, even equipment redesign can be considered, or some separators can be eliminated or used occasionally.

**Figure 10:**
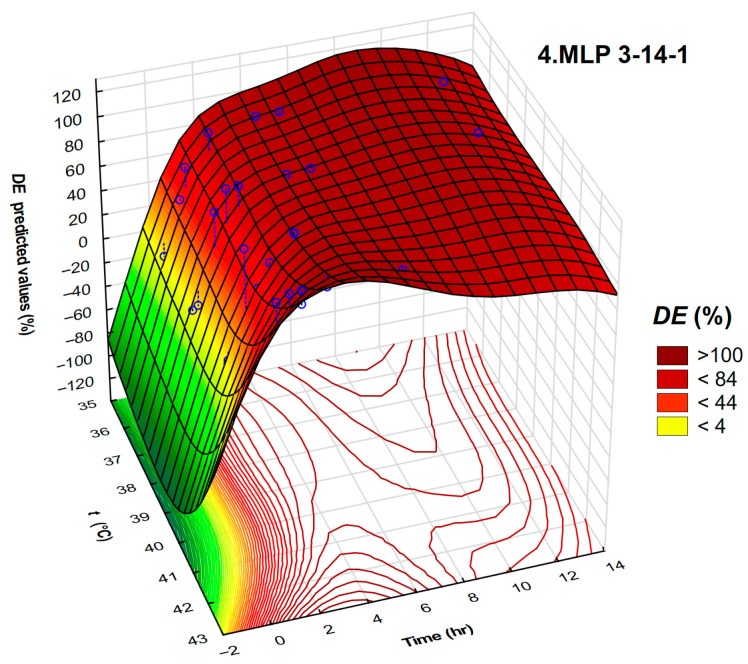
A three-dimensional plot representing the interdependence of demulsification efficiency, temperature, and time for demulsifier DEEM-5 and neural network 4.MLP 3-14-1 validation.

Interdependence of the demulsification efficiency, temperature, and time required for demulsification for DEEM-5 using neural network MLP 3-14-1 are given in Figure 10.

In the range of low time required for demulsification, *DE* is not influenced by the temperature. Demulsification efficiency sharply increases in the first hours of water settling over a whole range of temperatures. Higher values of demulsification efficiency are obtained after 2.5 h of settling time.

**Figure 11:**
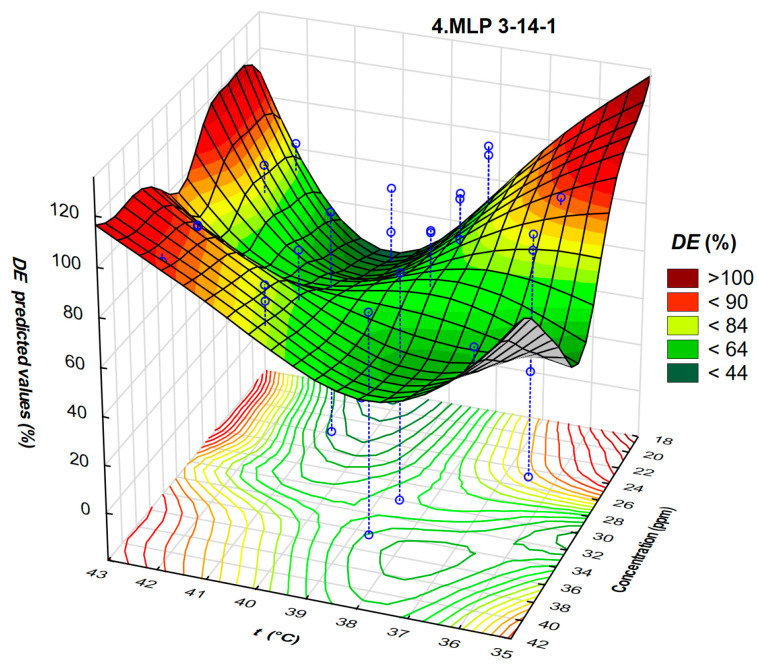
A three-dimensional plot representing the interdependence of demulsification efficiency, temperature, and concentration of the demulsifier DEEM-5 for neural network 4.MLP 3-14-1 validation.

Interdependence of DE, temperature, and concentration of the demulsifier DEEM-5 for neural network 4.MLP 3-14-1 is shown in Figure 11.

Four regions with high *DE* values are distributed at the edges of the 3D surface representing neural network results. The neural network approach achieves improved accuracy compared to the response surface analog (Figure 5b). This implies properly trained neural network indicating a well-fitting collection of data points.

Sensitivity analysis can help to select pertinent variables. The results of the sensitivity analysis are given in Table 7.

**Figure 12:**
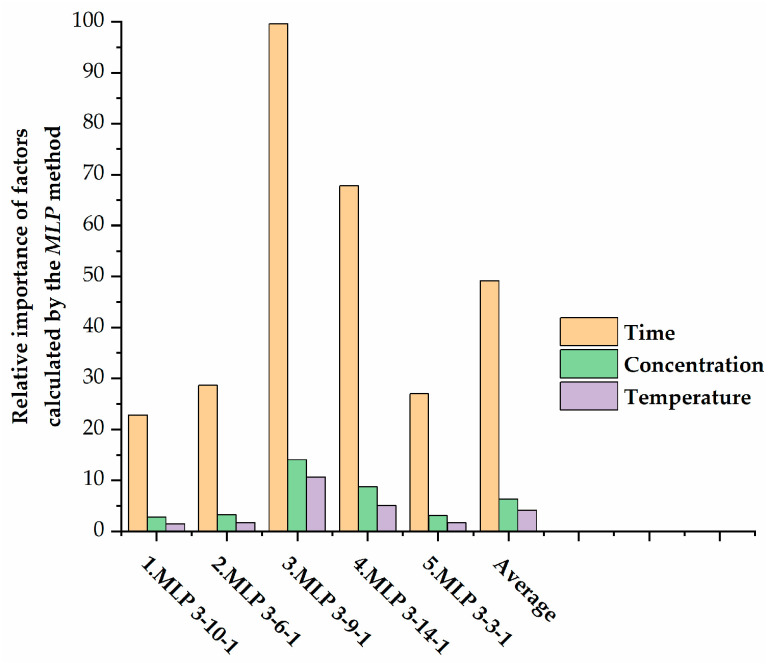
Relative importance of factors calculated by the MLP method.

Sensitivity analysis reveals the direct relationships of *DE* with time, concentration, and temperature. It is clear that time is the most important variable controlling demulsification efficiency, followed by demulsifier concentration and temperature (Figure 12).

From 3D plots and the results of sensitivity analysis, it can be concluded that a small increase in the time required for demulsification contributes to a higher demulsification efficiency.

In investigating the effect of different demulsifiers on demulsification efficiency, we kept the initial droplet size distribution, mixing shear rate, and water salinity constant. All five demulsifiers were tested on a single water-in-crude-oil emulsion. It is important to note that the nature of the crude oil can impact the demulsification efficiency, with factors such as initial droplet size distribution and salinity playing significant roles. However, preliminary field tests indicated that the correlations based on these single factors were weak. The multilayer perceptron model more effectively captures the combined nonlinear interactions than the quadratic response surface methodology. Consequently, further analysis will focus on the characteristics of the crude oil such as asphaltene content.

### 3.4. Effect of Crude Oil Nature on Demulsification Efficiency

Crude oil emulsion samples were collected from five different gathering stations. A total of 60 emulsion samples were collected and analyzed to determine the effect of crude oil nature on demulsification efficiency. Furthermore, the influence of asphaltene content on demulsification efficiency was analyzed. Asphaltene content and crude oil classification are given in Table 8.

The demulsifier DEEM-5 was selected as the most promising demuslifier that can break emulsions from all investigated gathering stations. This demulsifier was tested on crude oil emulsions from five different gathering stations. The processing conditions at each gathering station are given in Table 9.

**Figure 13:**
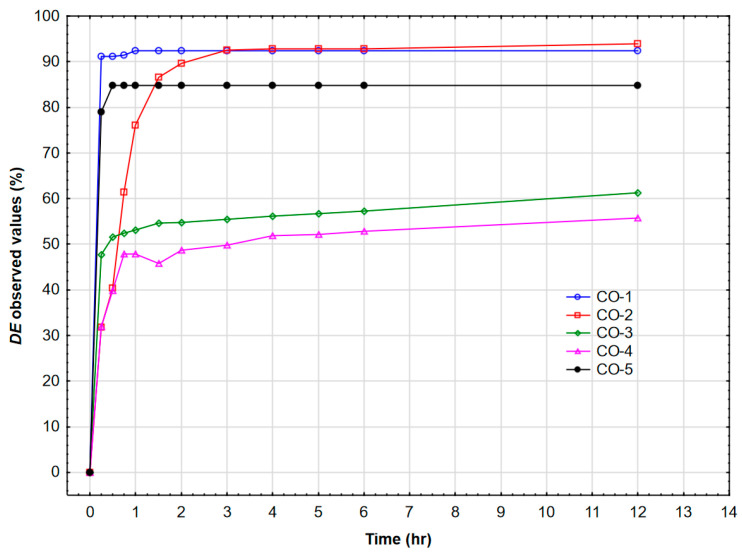
Demulsification efficiency of demulsifier DEEM-5 for different crude oils.

Figure 13 depicts the demulsification efficiency of demulsifier DEEM-5 for different crude oils. The highest demulsification efficiency was observed for crude oils CO-4 and CO-1. It should be noted that retention time is significantly shorter for the crude oil CO-1. The lowest demulsification efficiency was detected for the crude oil CO-4.

**Figure 14:**
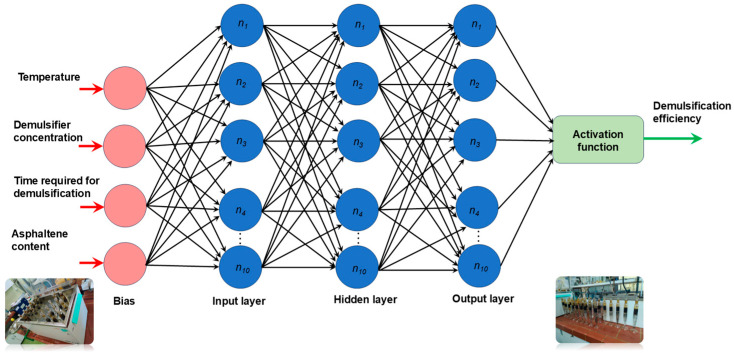
The architecture of automated neural network used for the investigation of the effect of temperature, demulsifier concentration, time required for demulsification, and asphaltene content on the efficiency of demulsification.

Figure 14 shows the architecture of automated neural network used for investigation of the effect of temperature, demulsifier concentration, time required for demulsification, and asphaltene content on the efficiency of demulsification. This approach was conducted to study and predict the demulsification efficiency of one demulsifier on different crude oils.

Random sampling was used as the sampling method, with 70% of data for training, 15% for testing, and 15% of data for validation. Furthermore, 1000 seeds were used for sampling. The number of hidden layers was from 3 to 10. Ten thousand networks were trained and evaluated based on sum of squares as error function on 60 samples of crude oil emulsions. A total of five neural networks with the lowest error function (sum of squares) were retained. A summary of selected automotive neural networks is shown in Table 10.

The highest validation performance is achieved with the neural networks MLP 4-7-1 and MLP 4-4-1. The neural network MLP 4-7-1 corresponds to the architecture of four input layers, seven hidden layers, and one output layer, while MLP 4-4-1 corresponds to the architecture of four input layers, four hidden layers, and one output layer.

A summary of active networks’ training algorithm and hidden and output activation is shown in Table 11. The neural networks MLP 4-7-1 and MLP 4-4-1 are developed by the hyperbolic tangent function for hidden and output activation.

**Figure 15:**
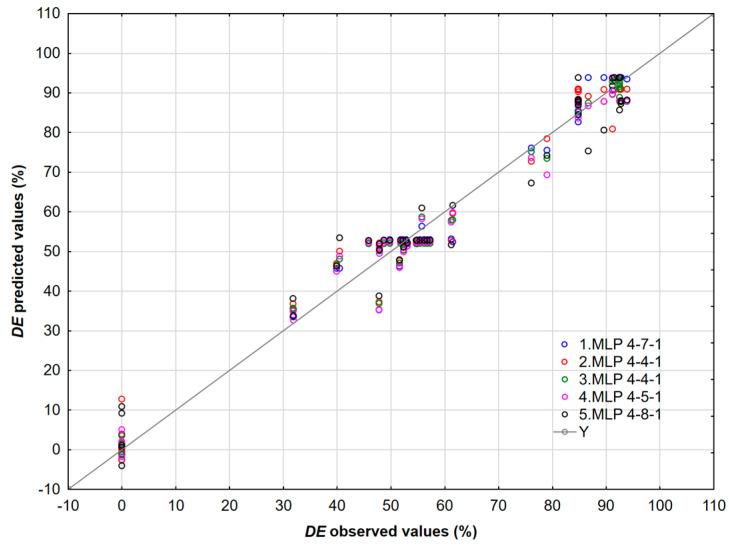
The relationship between the calculated and measured values of the *DE*.

**Figure 16:**
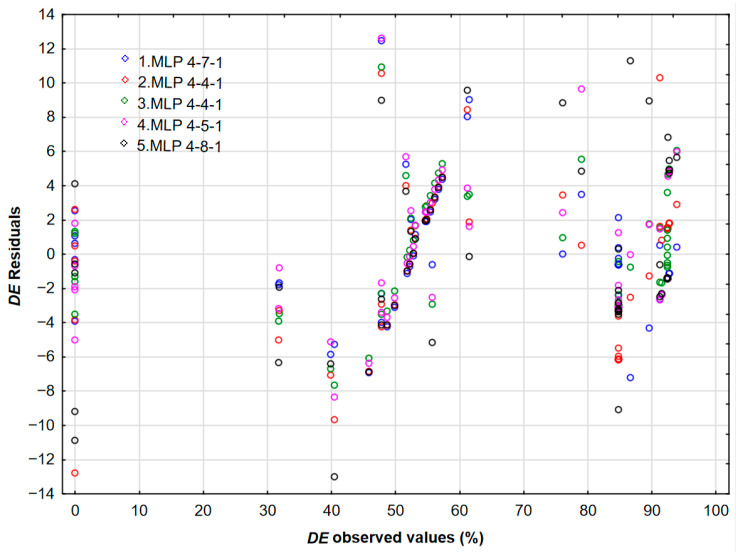
The relationship between the observed values of the *DE* and residuals.

The dependence of the predicted *DE* values on the experimental ones shows a good correlation (Figure 15), which is further confirmed by the interdependence of the observed values of the *DE* and residuals (Figure 16).

**Figure 17:**
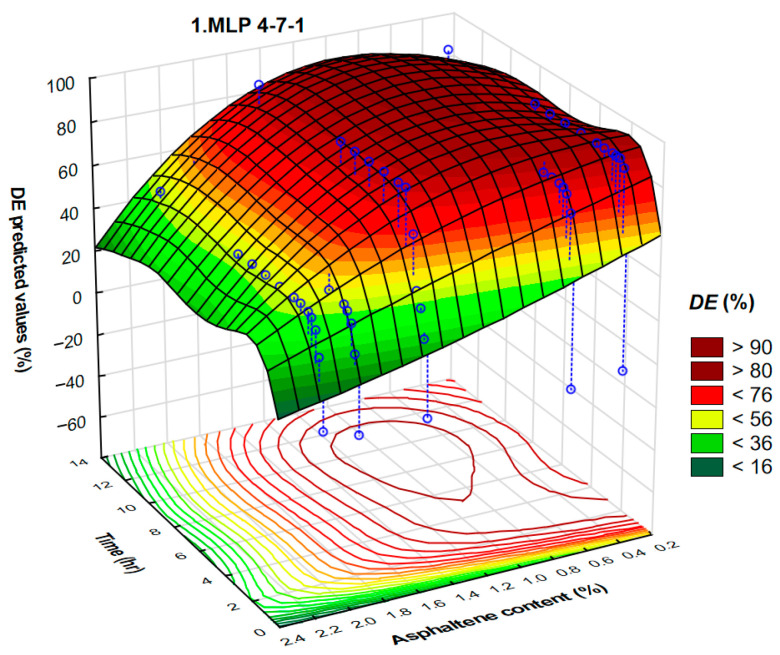
A three-dimensional plot representing the interdependence of demulsification efficiency, asphaltene content, and time of the demulsifier DEEM-5 for the neural network MLP 4-7-1.

**Figure 18:**
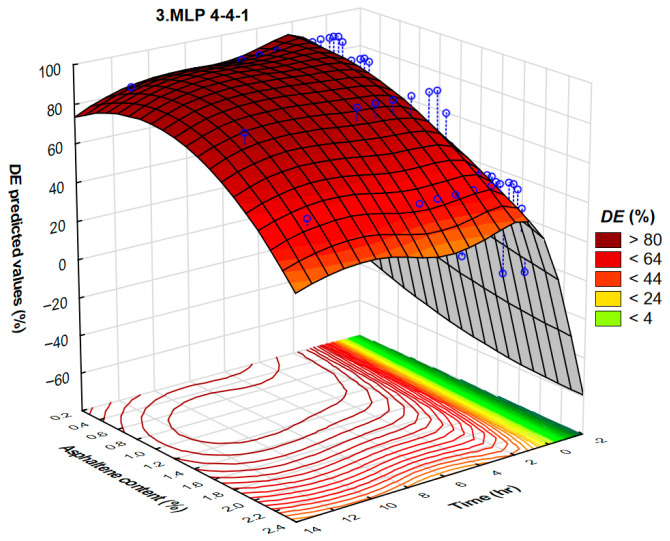
A three-dimensional plot representing the interdependence of demulsification efficiency, asphaltene content, and time of the demulsifier DEEM-5 for the neural network MLP 4-4-1.

Asphaltene content varied from 0.41 to 2.15%. It is well known that asphaltenes act as natural-occurring surfactants present in the crude oil. Due to the high asphaltene content, crude oils can act not only as Newtonian fluids but also as non-Newtonian (pseudoplastic or even Bingham’s) fluids. The results of automated neural networks and the interdependence of demulsification efficiency, asphaltene content, and time for MLP 4-7-1 and MLP 4-4-1 are presented in Figure 17 and Figure 18.

The 3D plots illustrate the regions of asphaltene content and time corresponding to the maximal demulsification efficiency (Figure 17). This is further confirmed by the contour diagram, developed by the projection of a 3D plot onto the XY plane (Figure 18). As far as we know, there are no published results in which such a systematic investigation was used to determine the crude oil demulsification working conditions. It is obvious that the highest values of demulsification efficiency correspond to the crude oils with lower asphaltene content.

The demulsification rate, defined as the percentage of free water released over time, is significantly influenced by several factors, including demulsifier nature, concentration, time required for demulsification, temperature, and the composition of the crude oil. Notably, DEEM-5 reached its highest efficiency of approximately 98% at a settling time of 4.5 h and a concentration of 36 ppm, as predicted by neural network models. This contrasts with the findings from the quadratic response surface methodology model, which suggested a longer settling time of 7.5 h and a concentration of 35 ppm. This acceleration in kinetics aligns with the research by Rondón et al. [15] and Borges et al. [16], who found that rapid droplet coalescence is facilitated by amphiphilic molecules that penetrate and disrupt rigid asphaltene films at the oil–water interface. Additionally, star-shaped surfactants have been shown to improve interfacial packing and curvature, further speeding up phase separation [1]. A global sensitivity analysis revealed that time, concentration, and temperature are the main factors affecting the demulsification rate, with time being the most significant. This pattern resembles findings from studies of derived demulsifiers [26] and green polymeric ionic liquids [20], where the tailored molecular structures impact film rigidity and micelle behavior. Overall, these results highlight that optimizing interfacial adsorption kinetics, as demonstrated through comprehensive factorial designs and advanced predictive modeling, is essential for achieving rapid and effective demulsification under field-relevant conditions.

## 4. Conclusions

The main aim of this investigation was to examine the effects of demulsifier nature, demulsifier concentration, temperature, and time required for demulsification on demulsification efficiency. The response surface method predicted the maximum of demulsification efficiency for 7.5 h and demulsifier concentration of 35 ppm, while the automated neural network approach shifted the optimum of demulsification efficiency to 4.5 h and demulsifier concentration to 36 ppm. The automated neural network MLP 3-14-1 corresponds to the architecture of 3 input layers, 14 hidden layers, and 1 output layer. The highest validation performance of 0.991932 is observed for the logistic output activation function and hyperbolic tangent hidden activation. The findings indicate that the neural network-based methodology effectively identifies regions with higher demulsification efficiency. This approach can serve as a promising and rapid screening tool for selecting reliable demulsifiers under a wide range of operating conditions. Additionally, the proposed statistical approach also simplifies the comparison of different demulsifiers. The analysis of the effect of crude oil nature on demulsification efficiency using neural networks confirmed the dominant influence of asphaltenes as natural-occurring surfactants. In this investigation, asphaltene content ranged from 0.41 to 2.15%. The highest validation performance is achieved with the neural networks MLP 4-7-1 and MLP 4-4-1. The neural network MLP 4-7-1 corresponds to the architecture of four input layers, seven hidden layers, and one output layer, while MLP 4-4-1 corresponds to the architecture of four input layers, four hidden layers, and one output layer. The highest validation performance of 0.996684 is achieved with the neural network MLP 4-7-1, while the validation performance of 0.994838 is achieved with MLP 4-4-1. The proposed ANN-based method offers a faster and more reliable way to predict demulsification efficiency, which can significantly cut down the cost of trial-and-error bottle field tests. This leads to substantial savings in chemical consumption. By identifying optimal conditions in shorter settling times, the method increases throughput and reduces residence time in separators, thereby enhancing energy efficiency and lowering utility demands, such as steam, heat, and pumping power. The scalability of the ANN approach is particularly important for large oilfields, where crude composition and operating conditions can vary greatly. Once trained, these models can be quickly adapted to new datasets, making them ideal for real-time decision support systems and integration into digital oilfield platforms. Overall, this work demonstrates that neural networks are not only a more accurate predictive tool but also a practical means of enabling sustainable, cost-effective, and scalable demulsification strategies in the oil and gas industry.

## Figures and Tables

**Table 1:** Crude oil properties.

Property	Value
Oil density at 15 °C, g∙cm^−3^	0.9181
API gravity at 15 °C	22.16
Kinematic viscosity at 20 °C, mm^2^∙s^−1^	127.2
Kinematic viscosity at 40 °C, mm^2^∙s^−1^	42.91
Pour point, °C	−31
Sulfur content, %	0.3
Molecular weight, g/mol	381.56
Saturates (wt %)	63.13
Aromatics (wt %)	22.65
Resins (wt %)	13.81
Asphaltenes (wt %)	0.41
Paraffin content (wt %)	7.1

**Table 2:** Demulsifiers type.

Demulsifier	Type
DEEM-1	alkoxylated block copolymer
DEEM-2	benzenesulfonic acid derivate
DEEM-3	glycerine alkoxylated
DEEM-4	phenolformaldehyde resin alkoxylated
DEEM-5	polypropylene glycol alkoxylated

**Table 3:** Test of sum of squares (SS) for whole model vs. residual.

Dependent Variable	Multiple *R*	Multiple *R*^2^	Adjusted *R*^2^	*F*	*p*
*DE*	0.810701	0.657236	0.648410	74.46137	0.00

**Table 4:** Activation functions for the hidden and output layer of neurons.

Activation Function	Function	Description
Identity	*a*	The neuron’s activation is delivered directly as the output.
Exponential	$e^{-a}$	The negative exponential function
Logistic sigmoid	$\frac{1}{1+e^{-a}}$	An S-shaped curve
Hyperbolic tangent	$\frac{e^{a}-e^{-a}}{e^{a}+e^{-a}}$	A sigmoid curve similar to the logistic function. It performs better than logistic function due to its symmetry, with improved multilayer perception of hidden layers.

**Table 5:** Summary of active networks for investigation of the effect of temperature, demulsifier concentration, and time on the efficiency of demulsification.

Index	Net. Name	Training perf.	Test perf.	Validation perf.
1	MLP 3-10-1	0.960641	0.989316	0.990772
2	MLP 3-6-1	0.964523	0.994383	0.991005
3	MLP 3-9-1	0.992609	0.996945	0.991174
4	MLP 3-14-1	0.987548	0.997346	0.991932
5	MLP 3-3-1	0.962225	0.994506	0.990792

**Table 6:** Summary of active networks’ training algorithm and hidden and output activation.

Index	Net. Name	Training Algorithm	Hidden Activation	Output Activation
1	MLP 3-10-1	BFGS 38	*Tanh*	*Exponential*
2	MLP 3-6-1	BFGS 48	*Logistic*	*Exponential*
3	MLP 3-9-1	BFGS 244	*Tanh*	*Logistic*
4	MLP 3-14-1	BFGS 64	*Tanh*	*Logistic*
5	MLP 3-3-1	BFGS 64	*Logistic*	*Exponential*

**Table 7:** Sensitivity analysis data.

Networks	Time	Concentration	Temperature
1.MLP 3-10-1	22.81960	2.77951	1.48014
2.MLP 3-6-1	28.61940	3.23402	1.70967
3.MLP 3-9-1	99.56704	14.03036	10.66468
4.MLP 3-14-1	67.77570	8.74096	5.04382
5.MLP 3-3-1	27.00319	3.08498	1.67510

**Table 8:** Asphaltene content and crude oil classification.

Crude Oil	Crude Oil Classification	Asphaltene Content (%)
CO-1	Naphthenic	0.41
CO-2	Paraffinic	1.56
CO-3	Paraffinic	2.15
CO-4	Paraffinic	1.95
CO-5	Paraffinic	0.72

**Table 9:** Gathering station conditions for demulsification.

Crude Oil	Demulsifier Concentration, ppm	Temperature, °C
CO-1	35	36
CO-2	52	50
CO-3	80	50
CO-4	80	50
CO-5	50	50

**Table 10:** Summary of active networks.

Index	Net. Name	Training perf.	Test perf.	Validation perf.
1	MLP 4-7-1	0.992811	0.980931	0.996684
2	MLP 4-4-1	0.987349	0.978165	0.990059
3	MLP 4-4-1	0.991780	0.982543	0.994838
4	MLP 4-5-1	0.991307	0.983771	0.990720
5	MLP 4-8-1	0.984221	0.972346	0.989044

**Table 11:** Summary of active networks’ training algorithm and hidden and output activation.

Index	Net. Name	Training Algorithm	Hidden Activation	Output Activation
1	MLP 4-7-1	BFGS 152	*Tanh*	*Tanh*
2	MLP 4-4-1	BFGS 113	*Logistic*	*Tanh*
3	MLP 4-4-1	BFGS 91	*Tanh*	*Tanh*
4	MLP 4-5-1	BFGS 131	*Tanh*	*Tanh*
5	MLP 4-8-1	BFGS 89	*Logistic*	*Tanh*

## Data Availability

The original contributions presented in this study are included in the article. Further inquiries can be directed to the corresponding author.
